# Providers’ preferences for pediatric oral health information in the electronic health record: a cross-sectional survey

**DOI:** 10.1186/s12887-017-0979-5

**Published:** 2018-01-11

**Authors:** Christopher M. Shea, Kea Turner, B. Alex White, Ye Zhu, R. Gary Rozier

**Affiliations:** 1 Department of Health Policy and Management, University of North Carolina at Chapel Hill, Gillings School of Global Public Health, Chapel Hill, NC USA; 2 Department of Dental Ecology, University of North Carolina at Chapel Hill, School of Dentistry, Chapel Hill, NC USA

**Keywords:** Electronic health record, Oral health, Dental health, Primary health care, Well child visit, Medicaid

## Abstract

**Background:**

The majority of primary care physicians support integration of children’s oral health promotion and disease prevention into their practices but can experience challenges integrating oral health services into their workflow. Most electronic health records (EHRs) in primary care settings do not include oral health information for pediatric patients. Therefore, it is important to understand providers’ preferences for oral health information within the EHR. The objectives of this study are to assess (1) the relative importance of various elements of pediatric oral health information for primary care providers to have in the EHR and (2) the extent to which practice and provider characteristics are associated with these information preferences.

**Methods:**

We surveyed a sample of primary care physicians who conducted Medicaid well-child visits in North Carolina from August – December 2013. Using descriptive statistics, we analyzed primary care physicians’ oral health information preferences relative to their information preferences for traditional preventive aspects of well-child visits. Furthermore, we analyzed associations between oral health information preferences and provider- and practice-level characteristics using an ordinary least squares regression model.

**Results:**

Fewer primary care providers reported that pediatric oral health information is “very important,” as compared to more traditional elements of primary care information, such as tracking immunizations. However, the majority of respondents reported some elements of oral health information as being very important. Also, we found positive associations between the percentage of well child visits in which oral health screenings and oral health referrals are performed and the reported importance of having pediatric oral health information in the EHR.

**Conclusions:**

Incorporating oral health information into the EHR may be desirable for providers, particularly those who perform oral health screenings and dental referrals.

## Background

Oral health is a key component of the overall health and well-being of children. Over the past two decades, the prevalence of dental caries has increased from 19% to 24% in children 2 to 4 years of age in the US [1]. Despite a high prevalence, dental caries often goes untreated in children under the age of 4 [2], which can cause pain and infections that interfere with eating, speaking, and learning [3]. Primary care physicians play a key role in the prevention of dental caries among young children through risk assessment, application of fluoride varnish, oral health education, and referrals to dentists, which can reduce future oral health expenses and improve long-term health outcomes [4–6].

The majority of primary care physicians support integration of children’s oral health promotion and disease prevention into their practices but can experience challenges integrating oral health services into their workflow [7, 8]. Recent studies suggest that including oral health information, such as oral health risk assessments and reminders for oral health referrals, in the electronic health record (EHR) can increase the provision of preventive oral health services in primary care. [9] [10] Although these initial results are promising, most EHRs in primary care settings do not include oral health information for pediatric patients [9, 10].

Recognizing the need to improve EHR design and use for supporting the care of children, a working group, funded by the Agency for Healthcare Research and Quality, continues to develop guidance for a children’s EHR format. The format includes the need for tracking provision of preventive services consistent with Bright Futures [11], such as oral health risk assessment, fluoride varnish applications, and dental referrals [12]. Given the various oral-health information elements that could be incorporated into primary care EHRs, it is important to prioritize the elements that would best support the service needs of children and the workflows of primary care providers.

Information systems theory and previous research suggest the importance of identifying user requirements [13] to help ensure that information is perceived as useful by providers [14–16]. The purpose of this study was to assess: (1) the importance of various elements of oral health information for pediatric primary care physicians to have in the EHR; (2) relative importance of the oral health information as compared to traditional elements of medical information for well-child visits; and (3) extent to which practice- and provider- characteristics are associated with EHR oral health information preferences.

## Methods

### Survey content and development

In an effort to increase the number of young children in North Carolina (NC) Medicaid who have a dental home, we disseminated a decision tool to improve oral health screening, risk assessment and referrals in medical offices. As part of the evaluation of this initiative [17], we developed a survey to assess primary care providers’ oral health promotion and disease prevention activities for infants and toddlers (children under the age of 4 years). Additionally, the survey examined the availability of EHRs for well-child visits, participation in meaningful use incentive programs, and provider information needs and preferences for oral health and other preventive services for well-child visits. We received Institutional Review Board approval from the University of North Carolina at Chapel Hill (IRB study #07–1942).

### Survey sample and administration

We surveyed primary care physicians in NC who provided care for Medicaid-enrolled children younger than 4 years of age from August – December 2013. Physicians who did not conduct well-child visits for this aged child, practiced in a tertiary academic health center or community clinic, or were not involved in any patient care were excluded from the study.

We developed the sampling frame using multiple sources of information including the National Plan and Provider Enumeration System [18], the NC Health Professions Data System [19], and NC Medicaid well-child visit data and Into the Mouths of Babes program participation records [20]. We verified the data and identified additional primary care practices and physicians by conducting online searches and making phone calls to practices. The final sampling frame included 1364 primary care physicians in 435 practices. We received a response from 50.3% or 219 of the 435 practices. We randomly selected one physician per practice to respond to the survey. If the selected physician did not respond, we randomly selected another physician from the same practice. We ensured that physicians who worked at multiple practices were surveyed only once.

We piloted the questionnaire with providers in 11 primary care practices participating in another study [21]. Sampled physicians were mailed up to three requests for participation via U.S. mail. To potentially reduce the non-response rate, we provided physicians with two options for completing the survey–a paper survey using a pre-paid envelope or an online survey developed using Qualtrics Survey Software (Provo, UT). Respondents were entered into a drawing for one of five Kindle Fire HD e-readers (a value of $200 at the time of survey administration).

### Practice characteristics

Prior studies have shown that practice characteristics, such as practice ownership, size, and urban location, affect primary care providers’ oral health activity for children [22–24]; therefore, we collected these data for our sample of providers. Practice ownership was coded as a categorical variable that included physician or physician group owned, academic medical center, non-academic affiliated hospital, and other. Practice size was measured as the number of physicians within the practice and was treated as a continuous variable. We transformed the zip code of the practice into a rural-urban commuting area code [25] and categorized the zip codes into urban and rural. Additionally, we included two binary variables including whether the practices used EHRs to conduct well-child visits and whether practices exclusively used an electronic system.

### Provider characteristics

We collected information on provider characteristics, including proportion of pediatric patients seen per week, oral health activities performed, and years since graduation from medical school. We hypothesized that the proportion of pediatric patients seen per week and the amount of oral health screening and dental referral activity would be positively associated with providers’ information preferences. We measured the proportion of pediatric patients as the ratio of pediatric patients (under age 4) to the total number of patients seen per week. We measured the amount of screening activity and oral health referral activity by asking physicians to estimate the percentage of all well-child visits (0%, 1–10%, 11–25%, 26–50%, 51–100%) in which they perform these activities. We also included years since graduation from medical school as a proxy for age because age is negatively associated with EHR adoption [26].

### Oral health information preferences

To assess providers’ oral health information preferences, we developed survey items based on the American Academy of Pediatrics’ clinical guidelines for infant and toddler oral health and recommendations from the U.S Preventive Services Task Force [27, 28]. Ten items assessed the importance (i.e., not important, somewhat important, or very important) providers place on an EHR containing oral health information for (1) risk assessment, such as listing risk factors for tooth decay; (2) intervention, such as listing prescriptions for fluoride supplements; and (3) referrals to a dentist.

To determine appropriateness of reducing any of the oral health information preferences survey items into a composite measure, we conducted a principal component analysis of the 10 items. We applied two decision rules to determine whether there was sufficient evidence for combining survey items into a composite index including a Kaiser-Meyer-Olkin Measure and a Bartlett’s Test of Sphericity [29]. We conducted a parallel analysis test to determine the number of factors to retain by comparing the observed eigenvalues extracted from the correlation matrix analyzed with those obtained from uncorrelated normal variables [30]. Based on the results, we retained one factor. We used factor scores from the principal components as weights, and a final oral health-information-preference composite index, ranging from 0 to 10, was constructed from the 10 items. The mean score was 7.13 (SD 2.19).

### Information preferences for non-dental preventive aspects of well-child visits

We asked providers about the importance of EHR information about other preventive aspects of well-child visits using the same 3-level response options as used for the oral health items. We developed these items based on recommendations from the American Academy of Pediatrics clinical guidelines for well-child visits [27],—specifically, how important it is for the EHR to plot growth charts and calculate height, weight, and body mass index (BMI); track adherence to well-child visits; track immunizations; calculate weight-based dosing; and calculate catch-up immunizations.

### Data analysis

We used descriptive statistics to assess information preferences for oral health and other preventive aspects of well-child visits. Furthermore, we analyzed associations between the oral health-information-preference composite index and key provider- and practice-level characteristics using an ordinary least squares regression model with bootstrapped standard errors. Since only one physician per practice was sampled, we assumed observations were independent and did not control for potential clustering effects. We ran three specifications of the model–one with a linear version of the dependent variable, one with a logarithmic version of the dependent variable, and one with the logarithmic version of the independent and dependent variables as a sensitivity analysis. We compared the results across the three models to ensure that estimates were robust and not sensitive to model specification. Since all three models produced similar estimates with the same level of statistical significance, we report the findings of the linear model for ease of interpretation. To assess whether missing values were missing at random, we compared the characteristics of individuals with and without missing data for the main variables of interest and did not find significant differences in characteristics. Therefore, we dropped missing cases from the model, reducing the sample size from 221 to 211. For these analyses, we used the statistical software Stata, version 13.0.

## Results

### Practice and provider characteristics

The analytical sample included 211 providers, 95.9% of sampled physicians. The majority of physicians worked in a practice owned by a physician or physician group (73.5%), and a practice located in an urban area (87.7%) (Table 1). Nearly 80% of physicians reported exclusively using an electronic EHR system for conducting well-child visits. On average, physicians worked in practices with 3.2 (SD 2.4) other physicians. Most physicians reported screening for oral health problems (89.6%) during at least half of well-child visits with infants and toddlers, and 51.2% reported making an oral health referral in at least half of well-child visits. The mean percentage of all patients seen per week who were infant or toddler was 48.0%.

**Table 1:** Practice and Provider Characteristics (*N* = 211)

Characteristics	Respondents N(%)
Practice ownership	
Physician or physician group	155 (73.5%)
Academic health center	21 (10.1%)
Hospital not affiliated with an academic health center	29 (13.7%)
Other	6 (2.8%)
Urbanicity	
Urban	185 (87.7%)
Rural	26 (12.3%)
Use of EHR for conducting well-child visits	
Yes, all electronic system	170 (80.6%)
Yes, part paper and part electronic	19 (9.0%)
No, but we plan to start using one within 12 months	14 (6.6%)
No, and we don’t plan to start using one within the next 12 months	8 (3.8%)
Percentage of well-child visits when provider makes oral health referral	
51–100% of visits	108 (51.2%)
26–50% of visits	48 (22.7%)
25–0% of visits	55 (26.1%)
Percentage of well-child visits when provider screens for oral health	
51–100% of visits	189 (89.6%)
26–50% of visits	18 (8.5%)
25–0% of visits	4 (1.9%)
Characteristics	Mean (SD)
Practice Size (number of physicians)	3.2 (2.4)
Years since graduation from medical school	20.4 (10.9)
Percentage of pediatric patients <4 years of age	47.8 (19.6)

### Oral health information preferences

Table 2 summarizes results about preferences for oral health information in the EHR. The largest percentage of physicians indicated that tracking topical fluoride applications was very important (69.2%). The smallest percentage of physicians indicated that providing test results for fluoride content of drinking water (31.3%) was very important.

**Table 2:** Summary of health information measures (N = 211)

Measures N(%)	Not Important	Somewhat Important	Very Important
Oral health information measures			
How important is it to you than an EHR/EMR system for young children…			
Track topical fluoride applications such as fluoride varnish	11(5.2%)	54 (25.6%)	146 (69.2%)
Record untreated tooth decay or other oral health problems	7 (3.3%)	63 (29.9%)	141 (66.8%)
List prescriptions for fluoride supplements	13 (6.2%)	63 (29.9%)	135 (64.0%)
Track referrals to a dentist	8 (3.7%)	79 (37.4%)	124 (58.8%)
Provide a link to patient oral health educational materials	9 (4.3%)	81 (38.4%)	121 (57.3%)
Provide reminders or prompts for guideline-based preventive oral health services	6 (2.8%)	85 (40.3%)	120 (56.9%)
Classify child’s oral health risk status based on a summary of risk factors	14 (14 (6.6%)	90 (42.7%)	107 (50.7%)
Contain information about the child’s dental home	12 (5.7%)	94 (44.5%)	105 (49.8%)
List individual risk factors for tooth decay	18 (8.5%)	112 (53.1%)	81 (38.4%)
Provide test results for fluoride in drinking water	50 (23.7%)	95 (45.0%)	66 (31.3%)
Other preventive well-child information measures			
Track immunizations	2 (0.9%)	10 (4.7%)	199 (94.3%)
Plot growth charts or automatically compute height, weight, and BMI percentiles	2 (0.9%)	11 (5.2%)	198 (93.8%)
Track adherence to recommended well-child visits	2 (0.9%)	20 (9.5%)	189 (89.6%)
Track catch-up immunizations	5 5 (2.3%)	35 (16.6%)	171 (81.0%)
Calculate weight-based dosing	10 (4.6%)	39 (18.5%)	162 (76.8%)

### Non-dental preventive well-child visit information preferences

Table 2 also summarizes preferences for having non-dental preventive well-child information in the EHR. The majority of physicians identified each of these elements as being very important, with the largest percentage of physicians indicating that tracking immunizations (94.3%) was very important and the lowest percentage indicating that calculating weight-based dosing (76.8) was very important. By comparison, this measure was rated as very important by more respondents than the highest-rated type of oral health information (tracking topical fluoride applications, 69.2%).

### Characteristics associated with oral health information preferences

Table 3 provides results for the regression model examining the association between the composite index scores and provider and practice characteristics. Among provider characteristics, percentage of pediatric patients, oral health referral activity, and oral health screening activity were significantly associated with oral health information preferences. Specifically, holding all else constant, a one percentage point increase in the percentage of toddler and infant patients was associated with an approximately 13.3 percentage point increase in the reported importance of oral health information in the EHR (*p* = 0.017). Compared to physicians who conducted oral health referrals in less than 25% of well-child visits, physicians who conducted oral health referrals in more than 51% were associated with a higher reported importance for oral health information in the EHR (*p* = 0.014). Similarly, physicians who conducted oral health screenings in more than 51% of well-child visits reported significantly higher importance for oral health information as compared to physicians who conducted oral health screenings in less than 25% of well-child visit (*p* = 0.013). We found that other provider characteristics, such as years since graduation from medical school and exclusive use of an EHR system for well-child visits were not significantly associated with oral health information preferences. Also, we did not find significant associations between oral health information preferences and practice characteristics, such as size, rural location, and ownership.

**Table 3:** Characteristics associated with oral health information preferences index scores

	*β* (SE)
Percentage of pediatric patients	1.92^**^ (0.73)
Oral health referrals	
Oral health referrals in less than 25% of visits	*(Reference)*
Oral health referrals in 26–50% of visits	0.29 (0.47)
Oral health referrals in 51–100% of visits	1.07^**^ (0.37)
Oral health screenings	
Oral health screenings in less than 25% of visits	*(Reference)*
Oral health screening in 26–50% of visits	0.82 (0.49)
Oral health screening in 51–100% of visits	1.39^**^ (0.47)
Years since graduation from medical school	−0.016 (0.013)
Practice ownership	
Physician or physician group	*(Reference)*
Academic health center	−0.86 (0.49)
Hospital not affiliated with academic health center	−0.580 (0.404)
Other practice types	−1.07 (0.44)
Rural practice	
Urban	*(Reference)*
Rural	0.06 (0.43)
EHR Use for Well-Child Visits	
Exclusive use of electronic EHR system – No	*(Reference)*
Exclusive use of electronic EHR system – Yes	0.19 (0.62)
Practice Size	0.082 (0.061)
_Constant term	4.72^***^ (0.92)
*N*	195
*R* ^*2*^	0.1825

**p<0.01, ***p<0.001

## Discussion

Our study assessed the relative importance that primary care physicians place on having specific elements of oral health information about young child patients in the EHR, as well as how their information preferences vary by practice and provider characteristics. In general, a lower percentage of primary care providers reported that pediatric oral health information is “very important,” as compared to more traditional elements of primary care information (e.g., tracking immunizations). However, a majority of providers perceived most of the oral health information items as being very important (7 of 10 items >50%). Furthermore, we found that the proportion of pediatric patients, the percentage of well child visits in which the physician performs dental screenings, and the percentage of well child visits in which the physician makes a dental referral all were positively associated with reported importance of having oral health information in the EHR.

Various guidelines and recommendations highlight the need for pediatric EHR systems that support oral health activities [31]. The Children’s EHR Format recommendations issued in 2013 [32] and the 2015 Priority List [11] require functional capability to report completion of recommended health supervision visits delivered according to the recommended periodicity of visits included in Bright Futures [4]. Unfortunately, most EHRs do not fully support pediatric well-child visits or related oral health activities [9, 31]. Research in NC and Pennsylvania found that it is difficult to engage EHR vendors in meeting the Children’s EHR Format requirements because they are not required for Meaningful Use [21, 33] and because the enhancements may not lead to an adequate return on investment [34]. This concern supports the notion that provider’s information preferences may be associated with the need for documentation and reporting of actions required for reimbursement and/or for local quality measures. If so, emphasizing oral health services in such measures could increase the impact of enhancing EHRs with oral health information.

Notably, our results suggest that providers may not want a substantial amount of oral health information. Instead, a small number of structured data elements may facilitate both the oral health screening and referral activity of these providers. For example, measures of untreated tooth decay or other oral health problems, topical applications of fluoride varnish, prescriptions for fluoride supplements, and dental referrals could enable providers to track oral health services and help ensure that the services are provided within appropriate time intervals. These enhancements could support the movement toward value-based care through the prevention of dental-related emergency department visits and expensive dental treatment services.

Although our study provides useful insight into provider information preferences, additional work may be needed to optimize the specific information elements and tools to be included in EHRs. For example, our results indicate a relative lower preference for classification of risk status, information about dental home, list of risk factors, and fluoride in drinking water, as compared to other items, such as tracking fluoride varnish applications and fluoride supplements, which appears contrary to previous findings that indicate EHRs should include validated screening tools to support recommendations from Bright Futures [11]. Future research could clarify further which specific information elements are highest priority, perhaps by comparing provider information preferences across multiple health care domains (e.g., oral health and mental health). Furthermore, future research could assess not only stated preferences for information elements but also actual use of the elements.

In addition to identifying priority information elements to include in the EHR, past studies have demonstrated, in other contexts, the importance of easy access to the information. For example, risk assessments for other childhood conditions, such as attention deficit disorder, are underutilized when the information is not presented within the well-child template [35]. Future studies should examine EHR design strategies to maximize ease of access to oral health information during well-child visits. Also important is determining how best to integrate oral health information collection into clinical workflows. For example, prior work suggests improving efficiency of risk assessment by collecting information from caregivers in the waiting room and automating the flow of data to the progress note [36]. To alleviate concerns about lack of time to perform oral health activities during a well-child visit, future research is needed to investigate such an approach to capturing oral health information, specifically, with minimal impact on workflow and patient waiting times.

### Limitations

This study was limited to Medicaid providers of services for children younger than 4 years of age in NC. Because NC was an early adopter of Medicaid reimbursement policies for preventive oral health services [37], NC-based physicians may have greater experience with oral health service delivery than physicians in other states, hindering the generalizability of our results. However, physicians with experience providing pediatric oral health services are better positioned to judge which elements of oral health information would be useful to support oral health screening and dental referral activity. Similarly, most of the practices in the sample were located in an urban area (87.7%), owned by a physician or physician groups (73.5%), and exclusively used EHRs for conducting well-child visits (80.6%). As a result, practice patterns and information preferences may not be generalizable to all primary care practices. Additionally, the survey did not collect information on availability of pediatric-specific information within the practice’s current EHR system, whichmay be an omitted variable from the OLS model. It is possible that preferences for oral health information could be a function of a providers’ current access to oral health information. In other words, the study could not identify whether the practices in the sample had protocols for oral health screenings, services, or referrals, and if documenting these activities was part of usual care. Omitting this variable could explain, at least in part, why our model did not account for more than 18% of the variation in practitioner responses. Nonetheless, this study makes a contribution to the literature by identifying primary care providers’ oral health information preferences in the EHR and provides evidence for future researchers to build upon.

## Conclusion

Primary care practices are being encouraged to provide services to promote oral health for children. Delivery of these services could be better supported by including pediatric oral health information in the EHR. Findings from this study suggest that specific elements of oral health information may be most useful, such as documenting topical fluoride applications, untreated tooth decay or other oral health problems, and prescriptions for fluoride supplements. Although our study is a first step toward identifying the priority elements of oral health information for primary care providers, future research is needed to validate our findings and identify whether additional oral-health information elements should be assessed.

## Abbreviations

BMI: Body Mass Index
EHR: Electronic health record
NC: North Carolina
SD: Standard deviation
